# Cerebral Venous Thrombosis in a Young Adult: Challenges in Diagnosing and Managing Risk Factors in a Resource‐Limited Setting

**DOI:** 10.1002/ccr3.73199

**Published:** 2026-07-19

**Authors:** Filmon Tesfay, Lidya Mussie, Samuel Tekle, Estifanos Habteab

**Affiliations:** ^1^ Keren Zonal Referral Hospital Keren Eritrea; ^2^ Afabet Hospital Afabet Eritrea; ^3^ Nakfa Hospital Nakfa Eritrea; ^4^ Tesseney Hospital Tesseney Eritrea

**Keywords:** case report, cerebral venous thrombosis, resource‐limited setting, smoking, young adult

## Abstract

Cerebral venous thrombosis should be suspected in young patients with severe headache and seizures, even without clear risk factors. In resource‐limited settings, early imaging and prompt anticoagulation can significantly improve outcomes despite incomplete thrombophilia workup.

**Abbreviations**
aPTT: activated partial thromboplastin time
CBC: complete blood count
CT: computed tomography
CVT: cerebral venous thrombosis
HEENT: head, eyes, ears, nose, and throat
ICSOL: intracranial space‐occupying lesions
INR: international normalized ratio
MRI: magnetic resonance imaging
PT: prothrombin time
UFH: unfractionated heparin

## 1 Introduction

Cerebral venous thrombosis (CVT) is clotting of blood in the sinuses of the skull on its way back to the heart after nourishing the brain [1]. It is a rare but important cause of stroke, particularly in young adults, accounting for approximately 0.5%–1% of all strokes worldwide [2]. Its clinical presentation is highly variable, often including headache, seizures, altered consciousness, focal neurological deficits, and signs of raised intracranial pressure [3]. The diversity in presentation can delay diagnosis, especially in the absence of classic risk factors or access to advanced imaging.

Risk factors include inherited and acquired thrombophilias, infections, malignancies, systemic inflammatory disorders, dehydration, pregnancy, hormonal contraceptives, smoking, and alcohol use, although in many cases no clear etiology is found [4, 5]. Yet, in low‐resource settings, assessment of many of these predisposing factors remains difficult due to limited access to advanced hematologic or immunologic testing. This often leaves clinicians reliant on history, basic labs, and clinical judgment.

Timely diagnosis and initiation of anticoagulation therapy are central to favorable outcomes in CVT [6]. It is well established that the current gold standard to depict CVT is the combination of conventional magnetic resonance imaging (MRI) with some kind of magnetic resonance venography, particularly with dynamic time‐resolved angiographic techniques, such as time‐resolved imaging of contrast kinetics (TRICKS) and time‐resolved imaging with stochastic trajectories. The TRICKS modality uses extremely rapid acquisitions to provide dynamic images of intravascular contrast flow. The use of TRICKS provides excellent spatial resolution, as well as dynamic flow information that has not been previously obtained without more invasive studies, such as interventional angiography [7, 8]. However, resource‐limited settings face challenges including limited access to advanced imaging and comprehensive thrombophilia screening, which complicates risk factor identification and management decisions [9].

This case report narrates the diagnostic journey and therapeutic challenges of managing a young male who presented with non‐specific neurological symptoms, in an environment constrained by limited diagnostics and treatment options. The case emphasizes both the diagnostic hurdles in risk factor assessment and the potential for favorable outcomes with guideline‐based management, even in low‐resource environments. This case also adds the importance of baseline imaging in navigating incidental findings and potential treatment complications.

## 2 Case History/Examination

A 24‐year‐old male patient, previously in relatively good health, presented with complaints of headache lasting 1 week and abnormal body movements of 2 days' duration. The headache began approximately 7 days prior and was initially mild during the first 4 days but progressively increased in intensity over the following 3 days. The patient described the headache as severe and unlike anything he had experienced before. It was localized primarily to the right side of the back head.

According to the patient's family, who accompanied him to the Emergency Department, he experienced four episodes of abnormal body movements in the last 2 days. These episodes were characterized by tonic contractions involving all four limbs accompanied by sustained gaze deviation toward one point. Each episode lasted between 10 and 20 min and was associated with loss of consciousness. The patient regained full consciousness between these episodes. He also experienced one episode of vomiting, which was non‐projectile, non‐bilious, and non‐bloody. Additionally, the patient complained of intolerance to light and sound, which were irritating to him during this period.

His past medical and social history revealed that he had been smoking about 10 cigarettes per day for 6 years and intermittently consumed alcohol in the form of beer and gin. There was no history of extremity weakness, fever, abnormal behavior, contact with a chronic cougher or known tuberculosis patient, or any other chronic illnesses such as diabetes mellitus or hypertension. There was no personal or family history of migraine headaches. There was no personal or family history of similar problems or hypercoagulable states.

On physical examination, the patient appeared acutely ill and was in severe pain. Vital signs at presentation were stable: pulse rate of 83 beats per minute, respiratory rate of 19 breaths per minute, blood pressure of 120/70 mmHg, and afebrile. Examination of the head, eyes, ears, nose, and throat (HEENT) showed pink conjunctiva, non‐icteric sclera, and pupils equal and reactive to light with normal size bilaterally. Chest, cardiovascular, and abdominal examinations were normal. Neurologically, the patient was conscious and alert with no meningeal signs or focal neurological deficits. Normal sensory and cranial nerve examinations.

## 3 Differential Diagnosis, Investigations and Treatment

Given the clinical presentation, the initial assessment in the Emergency department was migraine headache, with intracranial space‐occupying lesion (ICSOL) as a differential diagnosis. The patient was admitted to the emergency unit and managed symptomatically with diclofenac 75 mg intramuscularly as needed and ibuprofen 40 mg orally as needed. Laboratory investigations including complete blood count (CBC) were performed. Imaging with computed tomography (CT) of the head without contrast was scheduled during working hours.

Laboratory results showed hemoglobin of 16.92 g/dL, white blood cell count of 8.4 × 10 [3]/μL, hematocrit of 52.4%, platelet count of 242.6 × 10 [3]/μL, neutrophils 67.75%, and lymphocytes 23.11%. The CT scan performed the following day revealed cerebral venous thrombosis and left retro orbital cyst (Figure 1).

**FIGURE 1:**
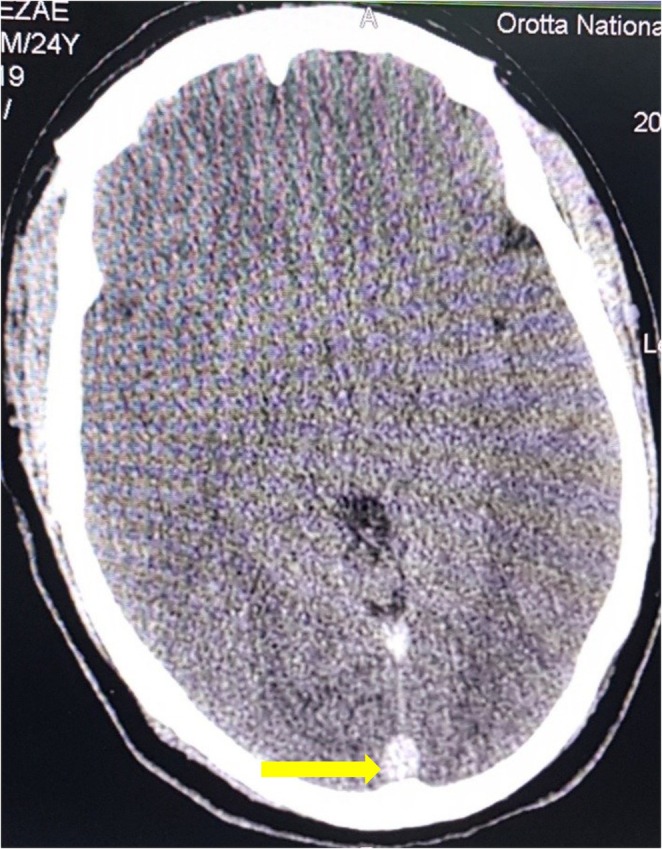
Non‐contrast computed tomography (NCCT) scan of the head at the level of the dural venous sinuses. The yellow arrow indicates a hyperdensity within the torcula herophili (confluence of sinuses), representing an acute venous thrombus.

Following the diagnosis, the patient was admitted to the medical ward and started on anticoagulation with unfractionated heparin (UFH) 5000 IU intravenously four times daily. Seizure prophylaxis with phenobarbital 60 mg orally once daily was initiated. Morphine was given for pain control, initially at 5 mg orally as needed and later increased to 10 mg after 2 days. Warfarin 5 mg orally once daily was started concurrently. Diclofenac was discontinued after 3 days. Laboratory monitoring included coagulation profiles (PT, aPTT, INR) and blood chemistry. Coagulation profile showed aPTT of 22.7 s (ref. range: 30–40 s), PT of 14 s (ref. range: 11–12.5 s), and INR of 1.343 (ref. range: 0.8–1.1). Blood chemistry revealed normal liver and kidney function parameters.

## 4 Conclusion and Results (Outcome and Follow‐Up)

The patient's headache began to improve gradually after 10 days of admission. While in the ward with the already mentioned management, the patient started to complain of left retro orbital pain. Magnetic resonance imaging (MRI) of the brain showed similar findings to the previous CT scan with no further intracranial finding (Figure 2). Both neurosurgery and ophthalmology specialists recommended conservative management. He was discharged on phenobarbital 60 mg once daily and warfarin 5 mg once daily. Coagulation profile at discharge revealed PT of 27.3 s, aPTT of 34 s, and INR of 2.72, indicating adequate anticoagulation. Warfarin was discontinued after 6 months of therapy. Unfortunately, detailed thrombophilia screening was unavailable due to limited laboratory resources, which is a common challenge in our setting. Only basic coagulation tests (PT, aPTT, INR) were accessible.

**FIGURE 2:**
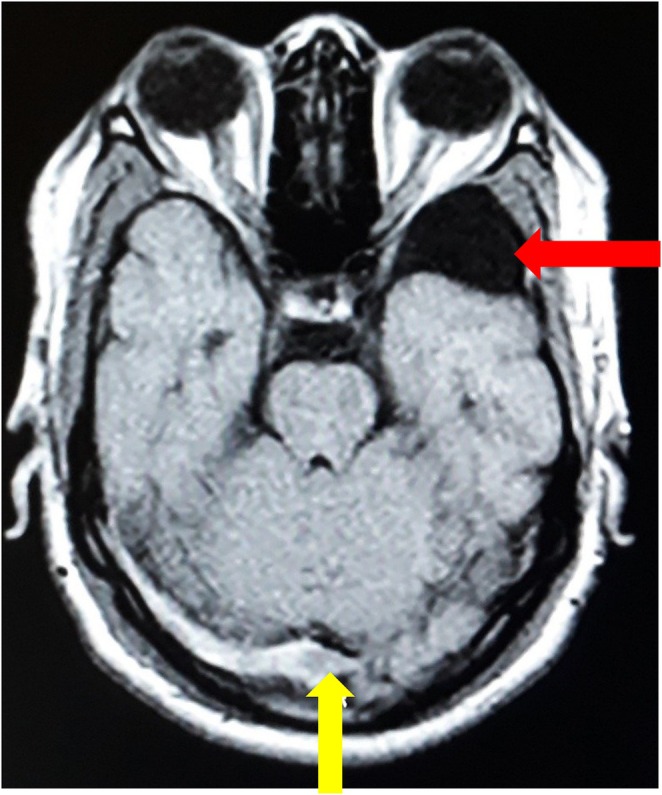
T1‐weighted (T1W1) axial magnetic resonance imaging (MRI) of the brain. The yellow arrow indicates a hyperintense thrombus in the right transverse sinus, consistent with venous thrombosis. The red arrow highlights the incidental arachnoid cyst in the left middle cranial fossa.

Subsequently, the patient experienced recurrent seizures, necessitating an increase in phenobarbital dosage to 60 mg twice daily. He has since been under regular follow‐up for seizure disorder.

This case underscores the complexities involved in diagnosing and managing cerebral venous thrombosis in young adults, particularly in low‐resource settings. While the diagnosis was confirmed with clear CT and MRI findings, the inability to perform extensive prothrombotic workup limited identification of underlying risk factors, reflecting common challenges in resource‐limited healthcare environments. The patient's history of smoking and intermittent alcohol use was noted but did not fully explain the etiology. Despite these constraints, timely initiation of anticoagulation with unfractionated heparin and warfarin, alongside seizure management with phenobarbital, contributed to clinical improvement and seizure control. This case emphasizes the importance of clinical vigilance, appropriate imaging, and adaptable management strategies in settings where diagnostic specialized tests are scarce. Furthermore, the identification of an arachnoid cyst in the left middle cranial fossa (congenital). This case highlights the need for improved access to comprehensive diagnostic tools and coordinated long‐term follow‐up to better understand risk factors and optimize care in similar contexts.

## 5 Discussion

Cerebral venous thrombosis (CVT) is a rare but significant cerebrovascular disorder, accounting for approximately 0.5%–1% of all strokes, predominantly affecting young adults [5]. The clinical manifestations of CVT are notably diverse and often nonspecific, ranging from headache to seizures, focal neurological deficits, or altered consciousness, making early diagnosis difficult [2]. In low‐resource settings such as ours, these challenges are further magnified due to limited diagnostic and laboratory capabilities.

Our patient presented with progressive severe headache and new‐onset seizures, symptoms that can mimic more common conditions like migraine or intracranial space‐occupying lesions (ICSOL) [3]. These differential diagnoses were initially considered, given the nonspecific clinical presentation, but were excluded following confirmatory neuroimaging demonstrating cerebral venous thrombosis. This highlights the crucial role of CT and MRI imaging in establishing diagnosis, especially when the image is (as in the present case) pathognomonic [10, 11].

A major diagnostic hurdle in this case was the inability to perform a comprehensive evaluation of prothrombotic risk factors. Although chronic smoking and intermittent alcohol consumption were identified as possible provocative factors to hypercoagulability [12], extensive thrombophilia workup, including genetic testing and assays for protein C, protein S, antithrombin III deficiencies, and antiphospholipid antibodies, was not available. The lack of these investigations is a common limitation in many low‐income countries and presents a significant barrier to fully understanding the etiology of CVT [4]. This absence of detailed risk factor profiling leads to a classification of many cases as idiopathic or unexplained, which complicates tailored management and prognosis estimation, as an underlying hereditary predisposition cannot be fully excluded, complicating decisions regarding the duration of long‐term anticoagulation [13]. To assist clinicians navigating similar diagnostic limitations, we have synthesized the common hypercoagulable states that require consideration in Table 1.

**TABLE 1:** Common hypercoagulable states in CVT.

Condition	Typical age of onset	Inheritance pattern	Clinical features/clues
Factor V Leiden	Young adulthood	Autosomal dominant	Most common genetic risk factor; often associated with venous thromboembolism (VTE).
Protein C/S deficiency	Neonatal to young adult	Autosomal dominant	Increased risk of skin necrosis (especially with warfarin initiation); recurrent VTE.
Antithrombin III deficiency	Young adulthood	Autosomal dominant	Resistance to heparin therapy; high risk during pregnancy/surgery.
Prothrombin G20210A mutation	Any age	Autosomal dominant	Elevated prothrombin levels; high prevalence in Caucasian populations (rare in African populations).
Antiphospholipid syndrome (APS)	20s to 40s	Acquired	Associated with arterial/venous clots and pregnancy complications; often seen in SLE.
Hyperhomocysteinemia	Any age	Genetic or nutritional	Linked to B12/Folate deficiency; increases both arterial and venous risk

Neurologically, the patient was alert without focal deficits or meningeal signs, consistent with the variable neurological presentations reported in CVT literature [14]. This subtle clinical picture further underscores why CVT can be easily overlooked in routine practice [15].

Therapeutically, the patient received unfractionated heparin transitioning to warfarin, consistent with current evidence supporting anticoagulation as the mainstay of CVT management regardless of hemorrhagic complications [6]. Phenobarbital was utilized for seizure control due to accessibility and affordability within our setting, although newer antiepileptic drugs might be preferred elsewhere [16]. Pain management and supportive care facilitated symptomatic relief, with notable clinical improvement by 10 days post‐admission. Initially, the patient's presentation was managed as a status migrainosus with NSAIDs. However, the lack of response to standard migraine therapy and the subsequent onset of seizures served as “red flags,” necessitating urgent neuroimaging. This highlights the importance of maintaining a high index of suspicion for CVT in young smokers presenting with refractory headaches, as early misdiagnosis is common and can delay life‐saving anticoagulation.

This case underlines the diagnostic and therapeutic challenges posed by CVT in low‐resource environments, particularly regarding comprehensive risk factor assessment and continuity of care. Improving access to advanced hematologic testing, neuroimaging, and coordinated follow‐up are crucial steps toward enhancing outcomes for patients with CVT in similar settings. Greater awareness of the clinical spectrum and pragmatic management strategies tailored to resource availability can help mitigate these hurdles [17].

The management of CVT in resource‐limited settings presents unique diagnostic and therapeutic challenges, particularly regarding the identification of underlying prothrombotic states. While standard guidelines recommend extensive screening for hereditary and acquired thrombophilia, the unavailability of such advanced testing in many developing regions necessitates a clinical approach focused on modifiable risk factors like smoking and dehydration [2]. Therapeutic choices also reflect resource availability; for instance, while newer anti‐seizure medications like levetiracetam are preferred for their lower side‐effect profile, phenobarbital remains a mainstay in low‐resource environments due to its high cost‐effectiveness and proven efficacy in managing refractory seizures [18, 19]. This case underscores the need for localized clinical algorithms that optimize available neuroimaging while navigating the limitations of laboratory infrastructure [20].

The primary limitation of this case study is the inability to perform a comprehensive thrombophilia workup, including assays for Protein C, Protein S, and Antithrombin III deficiencies, as well as genetic testing for Factor V Leiden. In Eritrea, such specialized laboratory tests are currently unavailable. Consequently, while smoking was identified as a provocative factor, a synergistic underlying hereditary predisposition cannot be entirely excluded. This underscores the necessity for developing regional centers capable of performing essential hematological screenings to better guide the duration of anticoagulation therapy.

## Author Contributions

**Estifanos Habteab:** writing – review and editing, methodology, investigation. **Samuel Tekle:** methodology, investigation, writing – review and editing. **Lidya Mussie:** writing – original draft, writing – review and editing. **Filmon Tesfay:** conceptualization, investigation, visualization, writing – original draft, writing – review and editing, methodology.

## Funding

The authors have nothing to report.

## Ethics Statement

The authors have nothing to report.

## Consent

Written informed consent was obtained from the patient for publication of this case report and utilization of any accompanying images.

## Conflicts of Interest

The authors declare no conflicts of interest.

## Data Availability

The data that support the findings of this study are available from the corresponding author upon reasonable request.
